# Supplementary material for: Identification of serum protein biomarkers in individuals with Niemann-Pick disease, type C1
Source: Biomark Res. 2026 May 9;14:63. doi: 10.1186/s40364-026-00927-x (PMC13307699; doi:10.1186/s40364-026-00927-x)
Supplement: Supplementary file 1 — Supplementary Material 1 [file 40364_2026_927_MOESM1_ESM.pptx]

## Slide 1
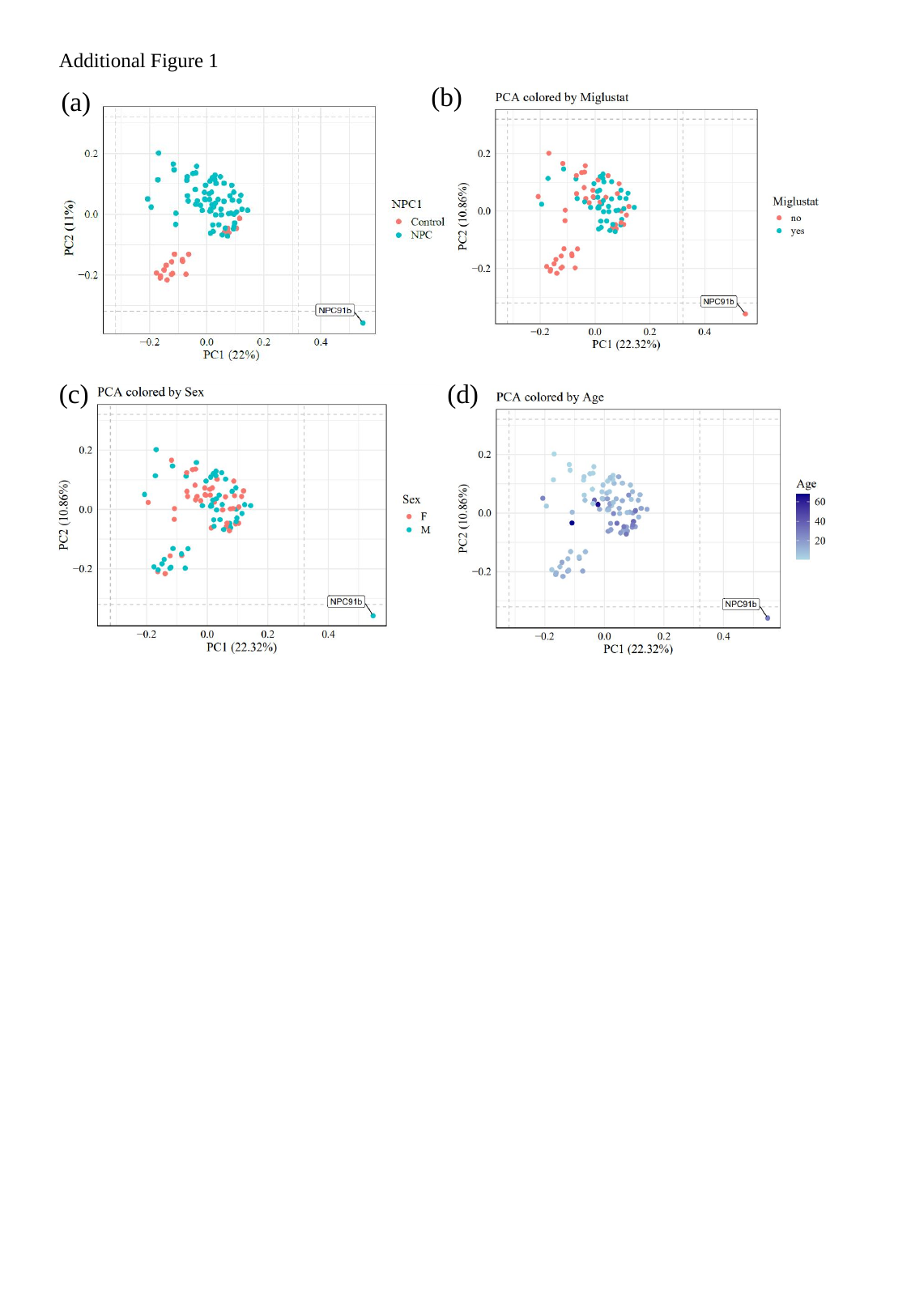

Additional Figure 1
(b)
(a)
(c)
(d)

## Slide 2
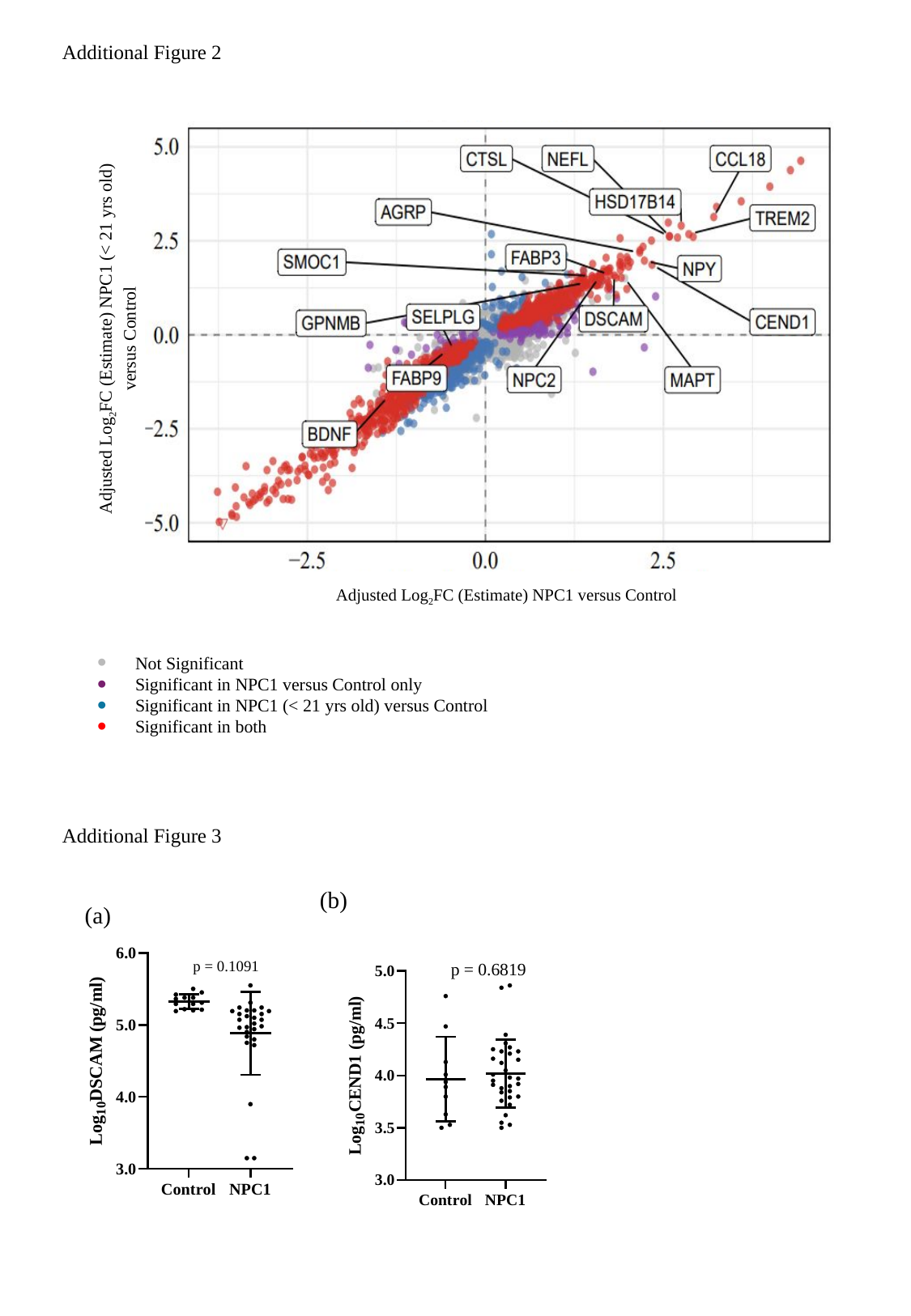

Additional Figure 2
Adjusted Log2FC (Estimate) NPC1 (< 21 yrs old) versus Control
Adjusted Log2FC (Estimate) NPC1 versus Control
Not Significant
Significant in NPC1 versus Control only
Significant in NPC1 (< 21 yrs old) versus Control
Significant in both
Additional Figure 3
(b)
(a)
p = 0.1091

## Slide 3
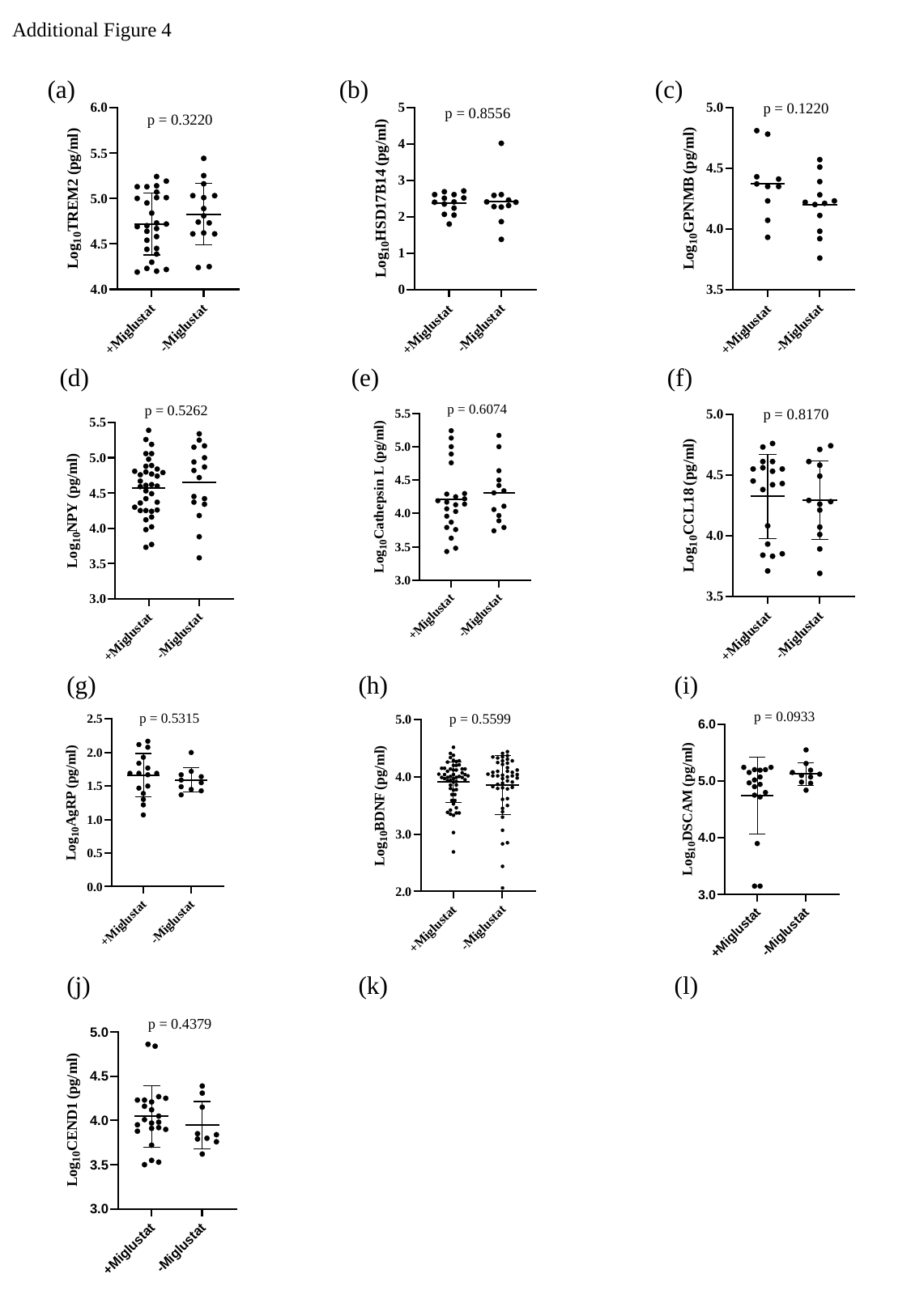

Additional Figure 4

## Slide 4
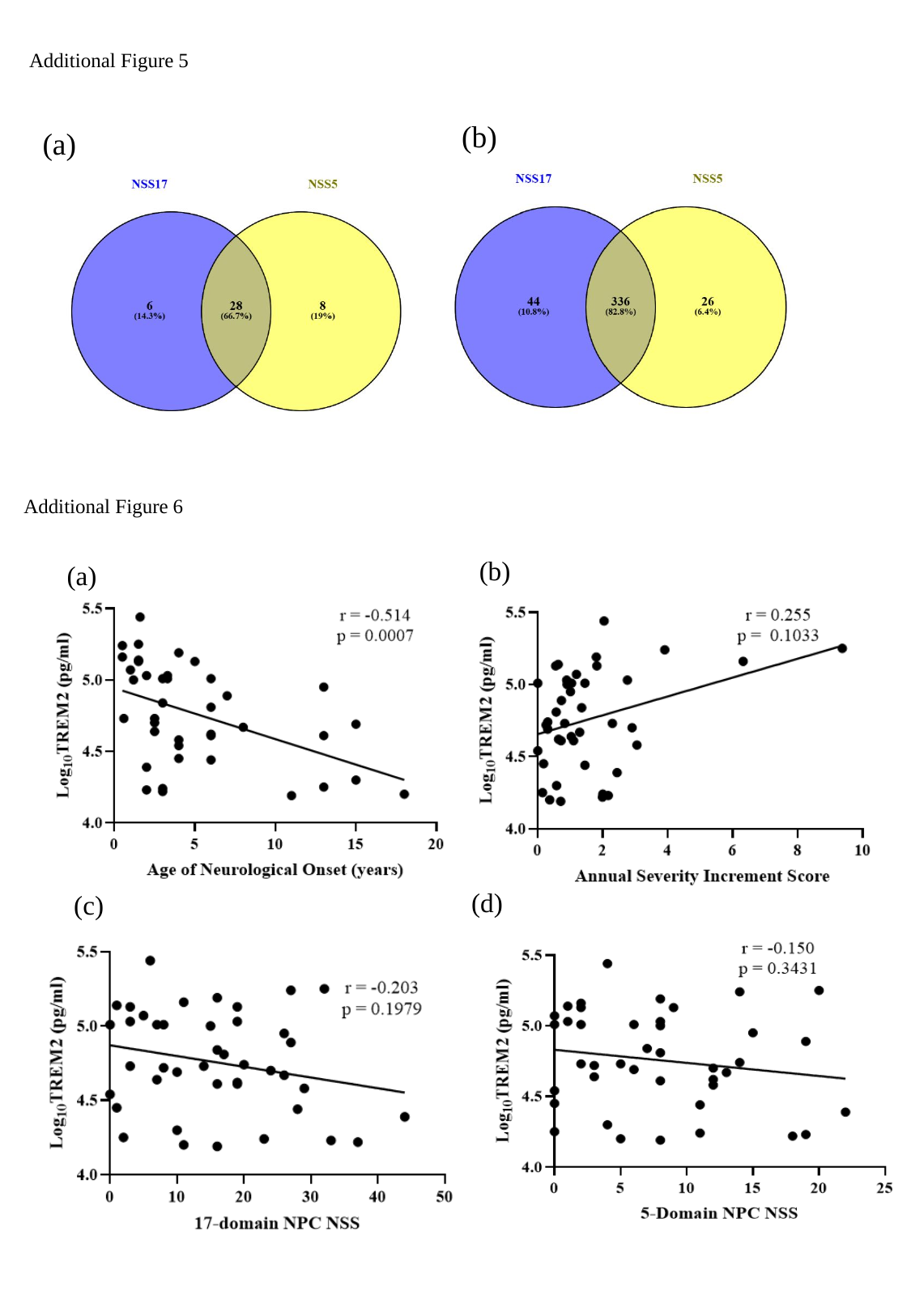

Additional Figure 5
(b)
(a)
Additional Figure 6
(b)
(a)
(d)
(c)

## Slide 5
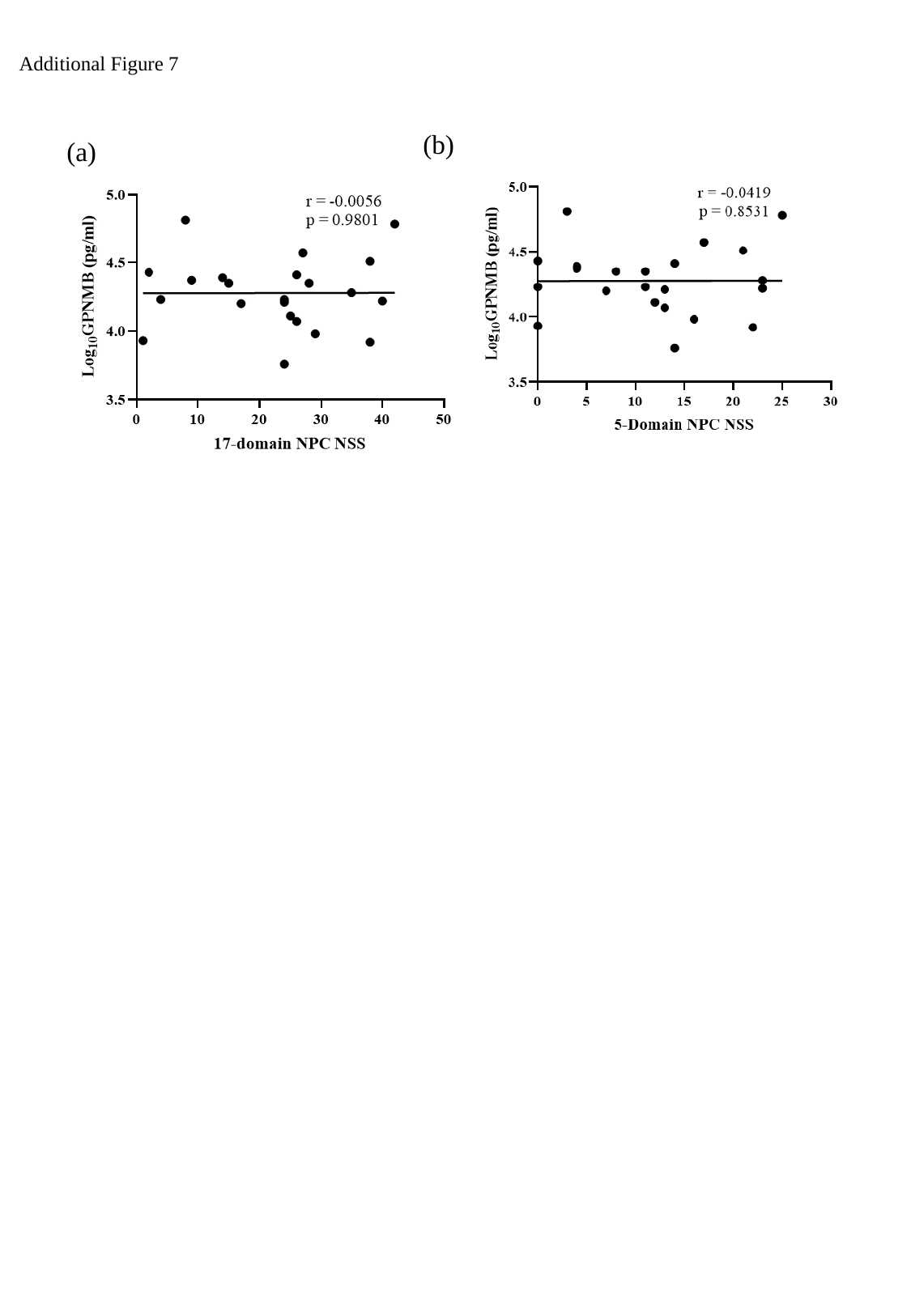

Additional Figure 7
(b)
(a)

## Slide 6
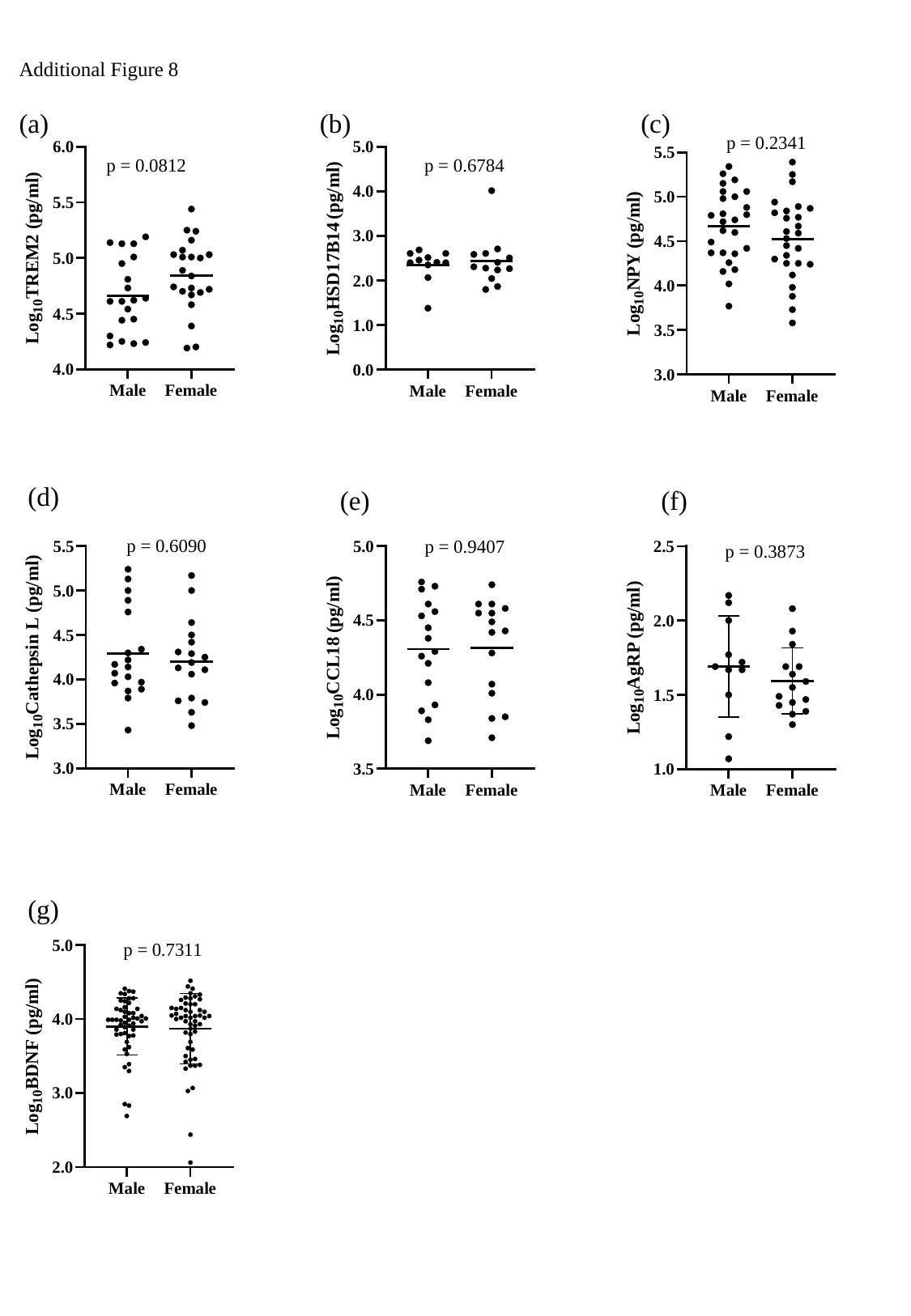

Additional Figure 8
(a)
(b)
(c)
(d)
(e)
(f)
(g)

## Slide 7
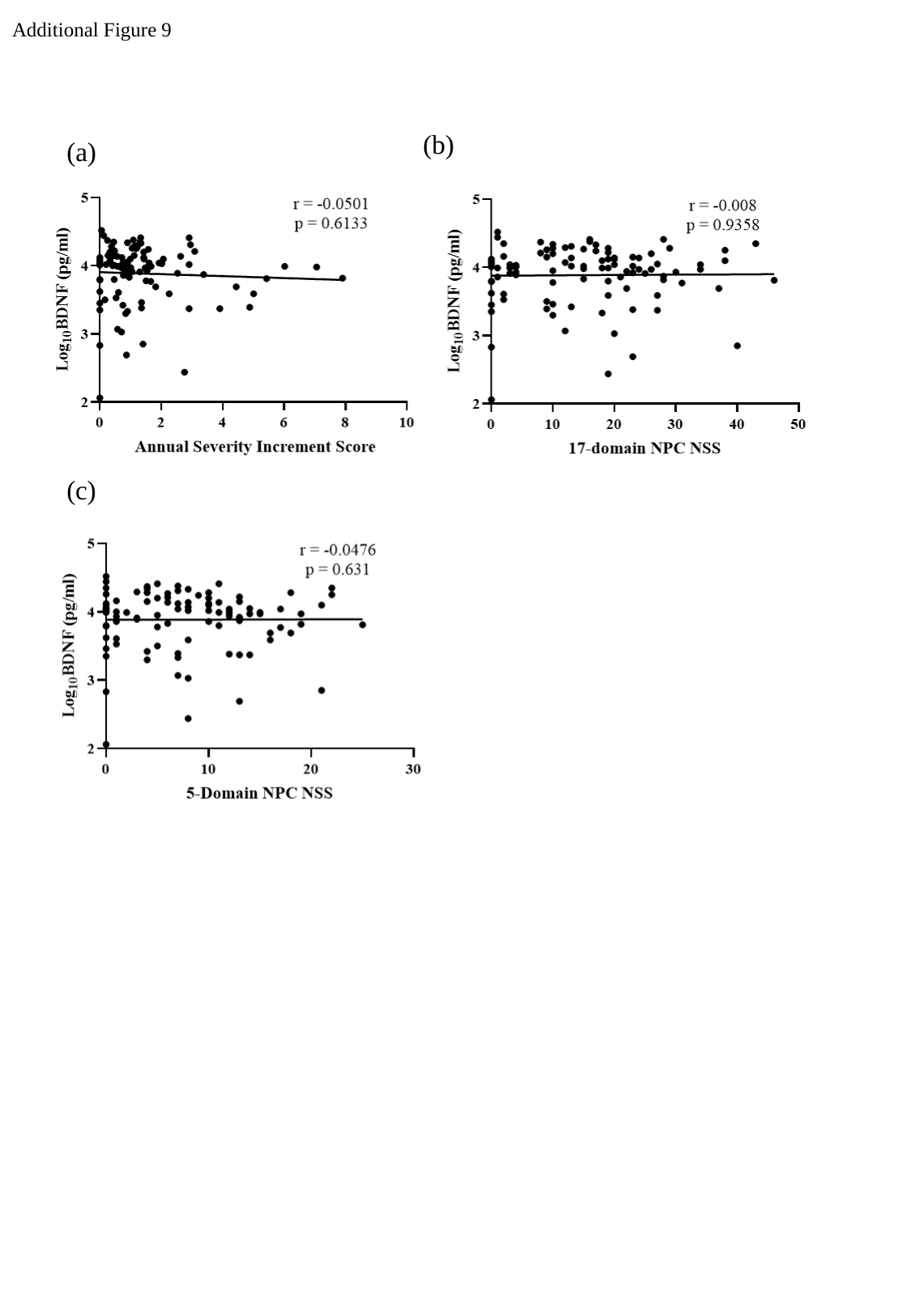

Additional Figure 9
(b)
(a)
(c)

## Slide 8
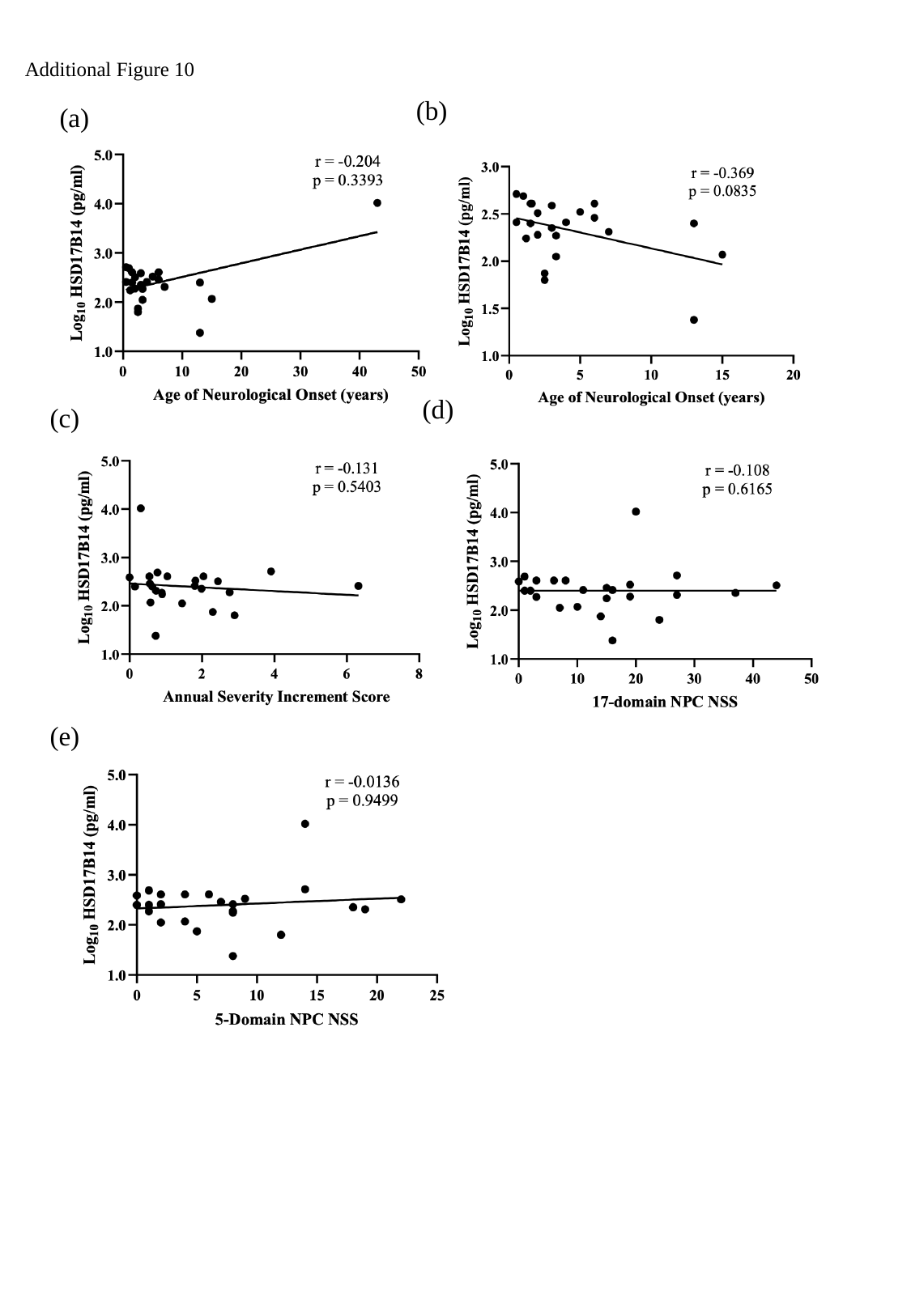

Additional Figure 10
(b)
(a)
(d)
(c)
(e)

## Slide 9
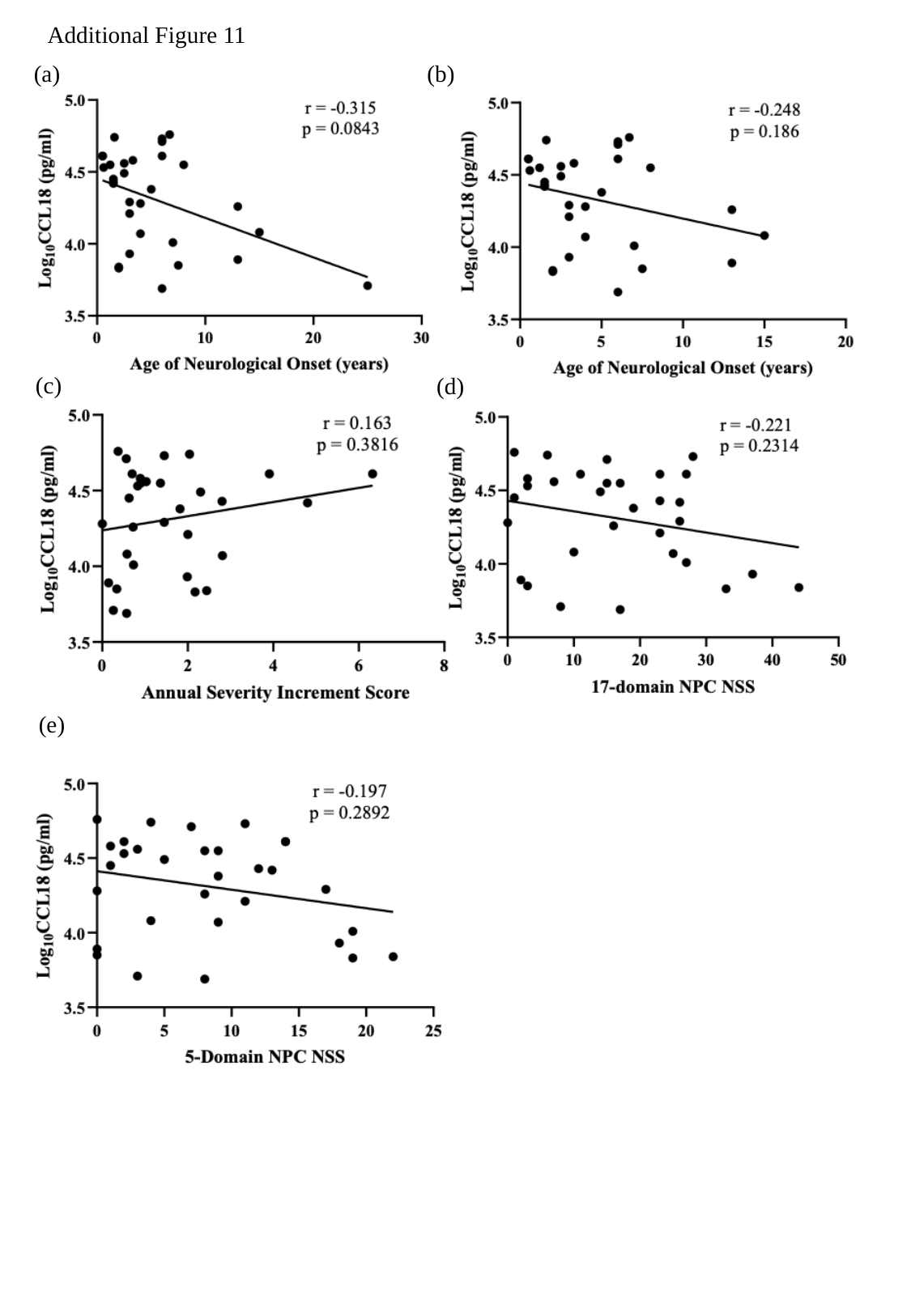

Additional Figure 11
(b)
(a)
(c)
(d)
(e)

## Slide 10
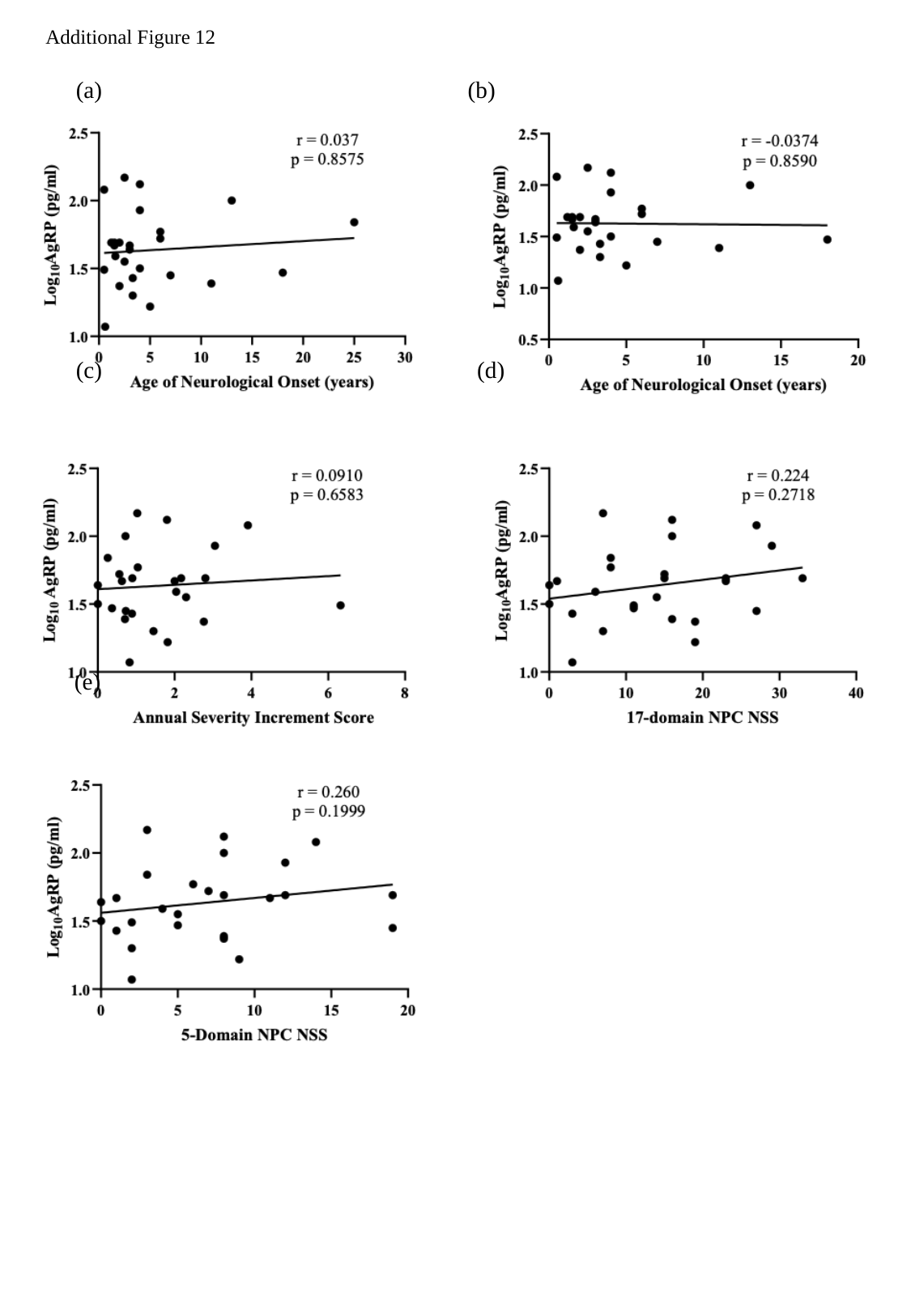

Additional Figure 12
(b)
(a)
(c)
(d)
(e)

## Slide 11
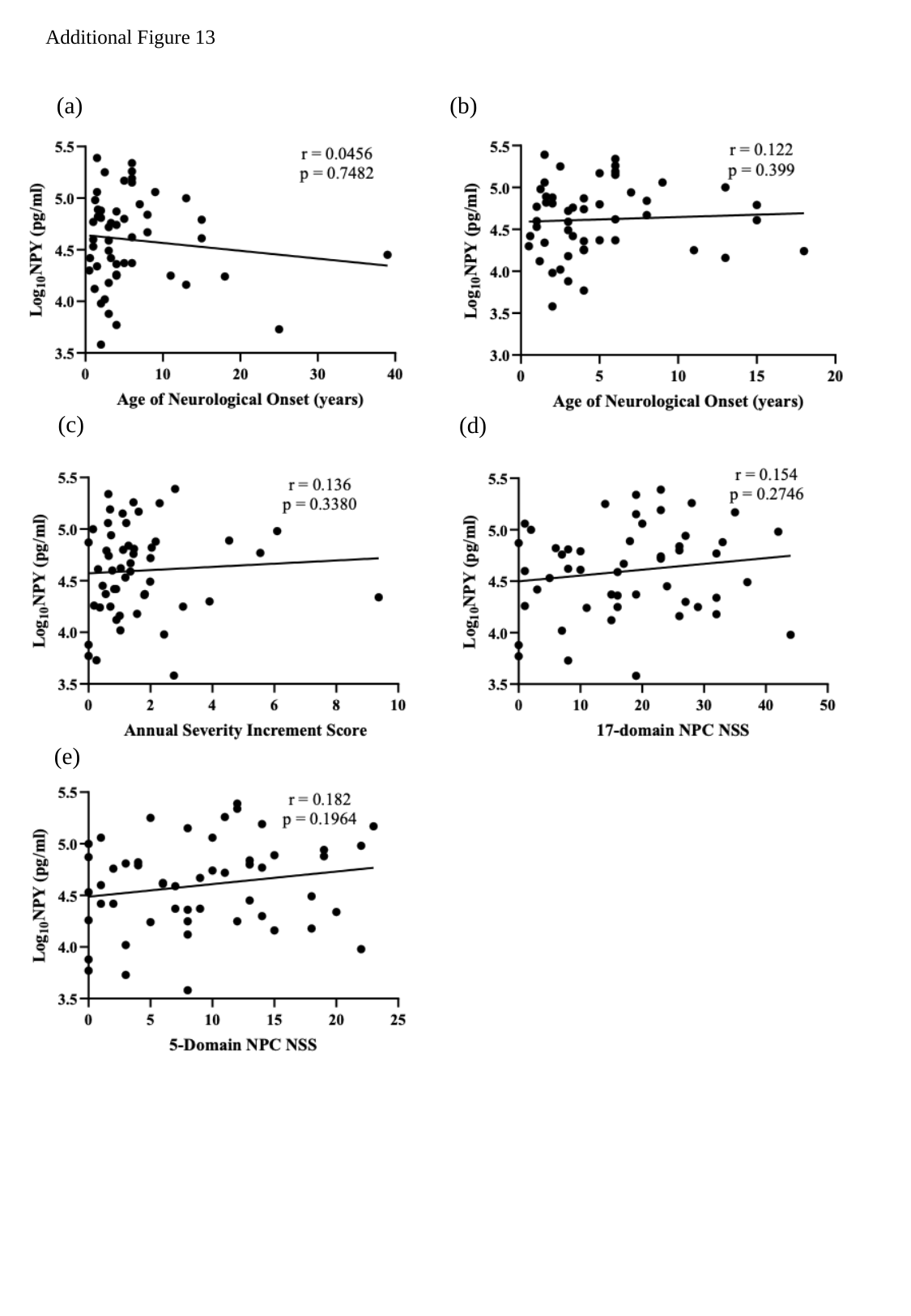

Additional Figure 13
(b)
(a)
(c)
(d)
(e)

## Slide 12
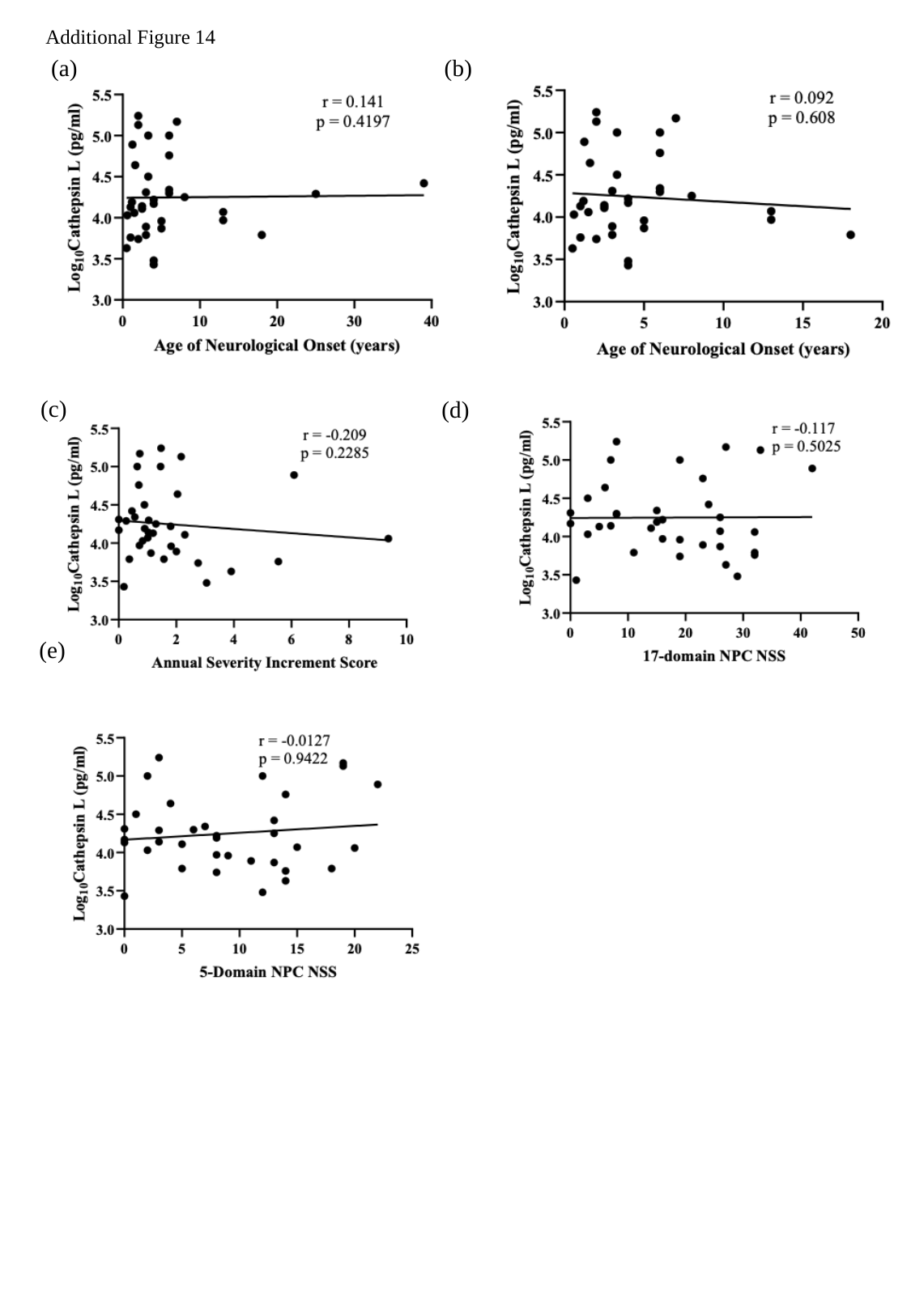

Additional Figure 14
(b)
(a)
(c)
(d)
(e)

## Slide 13
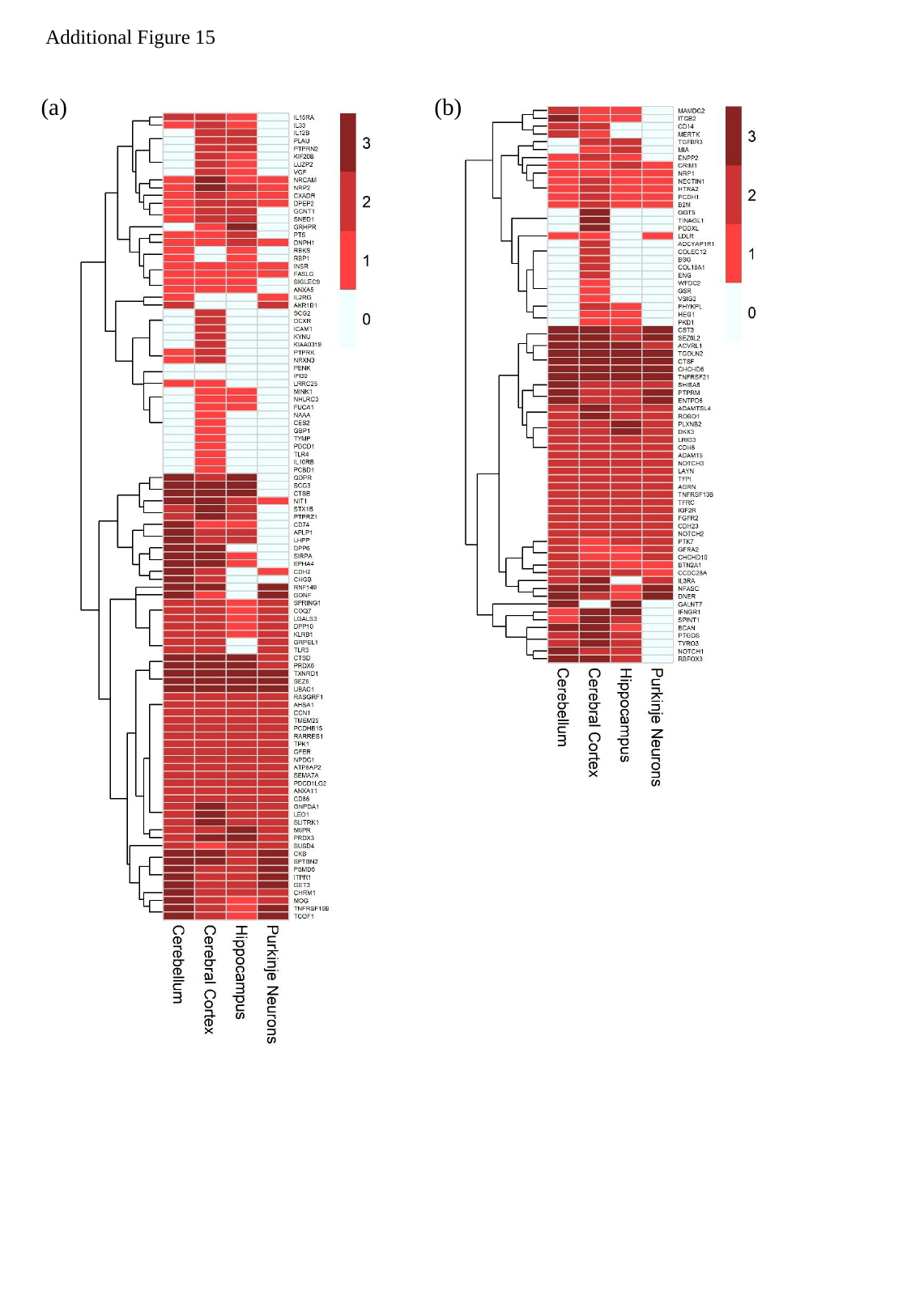

Additional Figure 15
(b)
(a)

## Slide 14
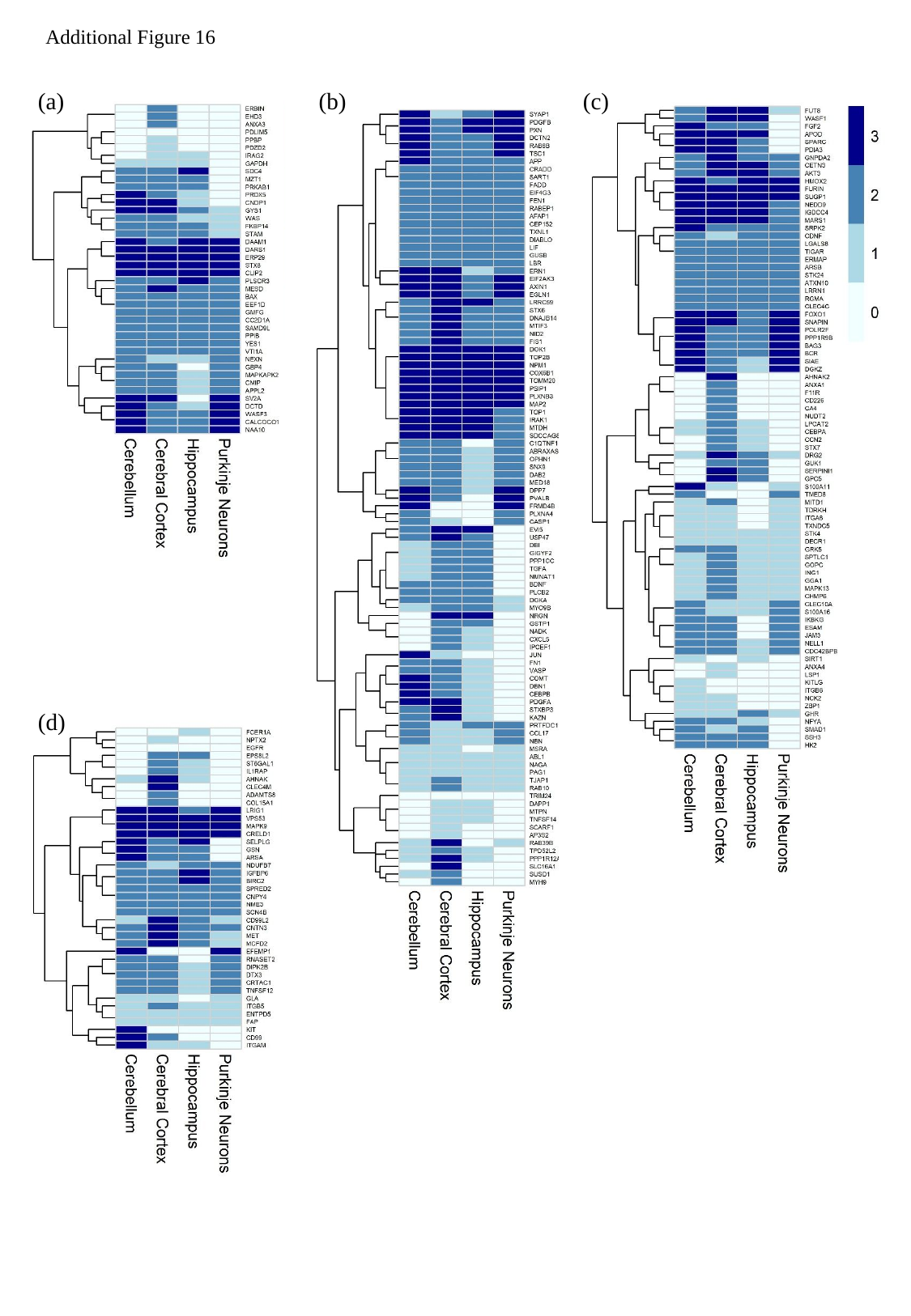

Additional Figure 16
(a)
(b)
(c)
(d)

## Slide 15
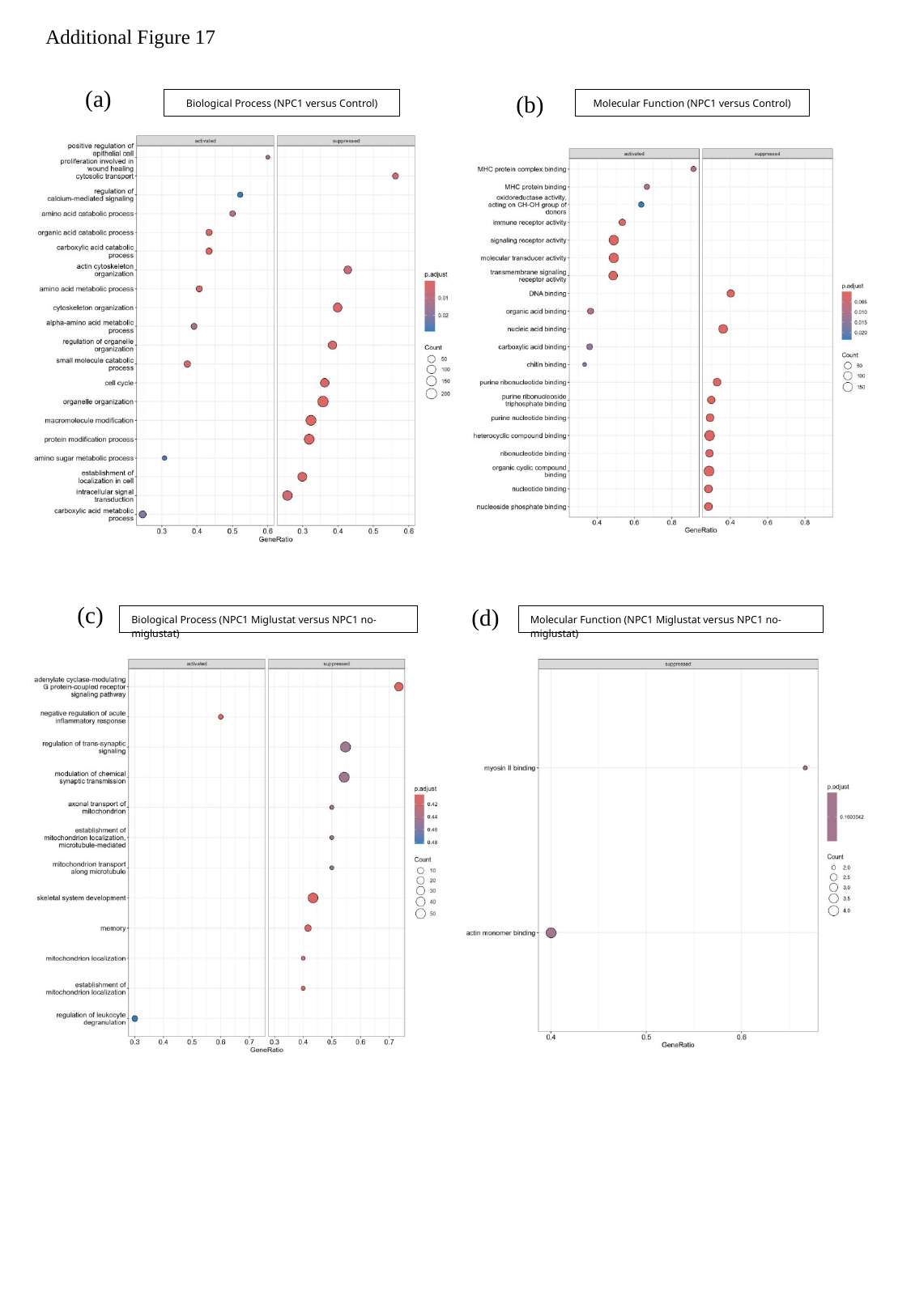

Additional Figure 17
(a)
(b)
Molecular Function (NPC1 versus Control)
Biological Process (NPC1 versus Control)
(c)
(d)
Biological Process (NPC1 Miglustat versus NPC1 no-miglustat)
Molecular Function (NPC1 Miglustat versus NPC1 no-miglustat)
