# Supplementary material for: Identification of serum protein biomarkers in individuals with Niemann-Pick disease, type C1
Source: Biomark Res. 2026 May 9;14:63. doi: 10.1186/s40364-026-00927-x (PMC13307699; doi:10.1186/s40364-026-00927-x)
Supplement: Supplementary file 2 — Supplementary Material 2 [file 40364_2026_927_MOESM2_ESM.docx]

## Additional Methods *Model diagnostics*

ANO and NSS17 were not used in NPC1 vs. control since these metrics were not captured for control samples. From our metadata correlations analysis (described below), we know age varies with NPX for 1,236/2,861 proteins at a rate of as much as +/-0.3 NPX per year of age. Our sensitivity analysis (described below) shows that age and NSS17 substantially affect our models (partial η²). ANO had a smaller partial η² than age or NSS17, and a strong enough effect to warrant the term’s inclusion in the miglustat-effect contrasts.

We examined between-group variance homogeneity with per-protein pairwise F-tests comparing our contrast arms. Heteroscedasticity was found in 5.6 % of proteins in the NPC1 vs. control contrast and 4.6 % in the miglustat treatment contrast (FDR < 0.10). The unequal-group variance assumption violation was discovered in a negligible proportion of our tests.

We assessed the normality of residuals (normal error) per protein with Shapiro–Wilk tests (Öztuna, 2006) adjusted by the Benjamini–Hochberg procedure (FDR < 0.10) and confirmed visually with quantile-quantile (QQ) plots. Residual sets with Shapiro–Wilk FDR < 0.10 showed heavy tails and some outliers. Heavy-tailed residuals were detected in 35 % of proteins in the NPC1 vs. control contrast and 43 % for the miglustat contrast.

We sought biomarkers that generalize across the heterogeneous NPC1 population. Classical parametric tests (e.g., ANOVA F-test) are known to become conservative when the error distribution contains biologically driven outliers. The realized Type I error is typically below the nominal α, while power is sacrificed as a tradeoff. This inherent conservatism aligns with our goal of minimizing false-positive protein discoveries in the presence of NPC1 patient-specific idiosyncrasies.

***Sensitivity analyses***

We used Cook’s distance heatmaps and leave-one-out (LOO) diagnostics to quantify the leverage of individual samples and assess each term's influence in our models. For every LOO iteration, we computed partial η² effect sizes with and without a sample or term and visualized the effect of removal with partial η² scatter plots. We inspected samples that appeared frequently or extremely in the heavy tails of the residual distributions. In the NPC1 vs. control contrast, samples showing the most considerable influence contributed to heavy-tail residuals for only a few percent of proteins. We observed similar heavy-tail patterns in the miglustat contrast, but no single sample exerted extreme influence ($\Delta$partial η²) enough to warrant exclusion.

***Covariate balance and metadata correlations***

We checked group comparability with Wilcoxon rank-sum tests for age and χ² tests for sex; no imbalance was detected (all BH-adjusted p ≥ 0.59). We calculated Spearman correlations between NPX, age, ANO, NSS5, NSS17, and ASIS for every protein, controlling the false-discovery rate at 0.10 (Additional Figure S1; Additional Table S7). Age NPX correlations testing used the full dataset containing all NPC1 and control samples ($n_{NPC1}$ = 67 &  $n_{control}$ = 20). Clinical variable NPX correlations testing used the NPC1 samples only, since these clinical variables are only recorded for NPC1 patients ($n_{NPC1}$ = 67).

Leave-one-out sensitivity analysis and correlation screening showed that age is a major driver: it correlated significantly with 1,173 proteins (FDR < 0.10) and strongly impacted partial η² in our sensitivity analysis. Sex showed lesser partial η² influence, but because sex distributions compared across contrast arms were not perfectly balanced and we have sex linked proteins in our data, we included sex in our models.

Disease severity metrics ANO, NSS5, and NSS17 showed substantial NPX correlations. To avoid over-parameterization in our miglustat effect contrasts, we chose the more granular NSS17 rather than including both NSS17 and the coarser NSS5 to allow due variance to be attributed to severity differences while detecting treatment effects. ASIS, defined as NSS17 divided by age, showed few correlations (1 at FDR < 0.10) and is intrinsically noisy and collinear with NSS17 and age and we therefore omitted ASIS as a covariate in all models.

**Additional Results**

**Evaluating the impact of missing adult controls**

We performed a sensitivity analysis comparing the original NPC1 vs control contrast with a younger version of the same analysis including NPC1 individuals less than 21 years of age. The new contrast removes adult NPC1 samples so that the comparison is restricted to young NPC1 individuals vs controls.

| Comparison group | NPC1 | Control |
| --- | --- | --- |
| NPC1 v/s Control (original analysis) | 24 | 20 |
| NPC1 under 21 yrs v/s Control (New analysis) | 19 | 20 |

This comparison evaluates whether the disease signal identified in the full analysis remains stable when restricting the dataset to pediatric samples.

The adjusted fold-changes are highly concordant across proteins between the two analyses. The proteins that were selected for the validation and are discussed in the manuscript were also significant in both the analysis. This indicates that the major disease-associated signals are largely preserved when restricting the analysis to pediatric samples. We conclude that our original NPC1 versus Control model does a good job at extrapolating adult control NPX levels and is robust to the missing data.

*Partial η² (pes): The proportion of total variance in the response (NPX) that is uniquely attributable to a given model term, after accounting for all other terms. It ranges from 0 (no variance explained) to 1 (all variance explained).*

**Functional Enrichment of the differentially abundant proteins**

We performed gene set enrichment (GSEA) across different contrasts (NPC1 versus Control and NPC1 miglustat versus NPC1 not receiving miglustat). We used GSEA because 1) it does not require a threshold and 2) the algorithm naturally does not require a separate background list to compare against. This latter point is important, since we do not have whole proteome and instead a specific set of panels with a priori functions (e.g., the Neurology panel we expect to be enriched in neuron-related proteins). We have used adjusted p-values using the Benjamini-Hochberg method.

While comparing NPC1 versus Control proteins, upregulated proteins (activated) were enriched in biological processes (BP) such as regulation of epithelial cells proliferation involved in wound healing, calcium-mediated signaling, catabolic and metabolic processes and molecular function (MF) related to MHC protein binding, immune receptor activity, signaling receptor activity. This suggests activation of immune system function, especially antigen presentation and receptor mediated signaling pathways. Whereas downregulated proteins (suppressed) were enriched in biological processes such as cytosolic transport, cytoskeleton organization, cell cycle, and protein modification. Molecular function of downregulated genes included DNA binding, nucleic acid binding, purine-ribonucleotide binding. This suggested suppression of gene regulatory mechanism and basic cellular biosynthetic activity in NPC1.

Comparison of differentially abundant proteins in miglustat versus no miglustat treated samples revealed enrichment of biological processes: negative regulation of acute inflammatory response and regulation of leukocyte degranulation for upregulated proteins (activated) in miglustat treated samples. Decreased proteins (suppressed) were enriched in biological processes related to GPCR signaling, synaptic transmission, mitochondria localization, memory, and molecular function related to myosin-II binding and actin monomer binding, suggesting suppression of cellular respiration and synaptic signaling.

Although this analysis provides insight into potentially disrupted pathways and processes, it does not capture the full spectrum of disease-related alterations given the limited number of proteins assessed (~1000) relative to the complexity of the serum proteome.

**Legends Additional Figures and Tables**

**Additional Figures**

Additional Figure 1: PCA plot based on NPX values to examine the clustering of samples and identify any potential outliers. PCA plot colored by (a) disease status, (b) miglustat status, (c) Sex, and (d) Age. Only one NPC1 individual, NPC91b, a 29.7-year-old male, was an outlier and therefore removed from the downstream analysis.

Additional Figure 2: Evaluating the impact of missing adult controls: Integration of differentially abundant serum proteins in NPC1 disease versus control, on the X-axis, and NPC1 (<21years old) versus controls on the Y-axis. Proteins discussed in the manuscript are labelled.

Additional Figure 3: ELISA measurements from NPC1 and control serum samples (a) DSCAM levels, and (b) CEND1 levels.

Additional Figure 4: Expression levels of proteins based on miglustat treatment ELISA assays: there were no differences in the expression level of (a) TREM2, (b) HSD17B14, (c) GPNMB, (d) NPY, (e) Cathepsin L, (f) CCL18, (g) AgRP, (h) BDNF, (i) DSCAM, and (j) CEND1. An unpaired two-tailed t-test was used to evaluate the differences between miglustat-treated and untreated samples.

Additional Figure 5: Overlapping protein correlations between NSS17 and NSS5 (a) Overlap between positively correlated proteins, and (b) Overlap between negatively correlated proteins

Additional Figure 6: Spearman correlation of NPC1 serum TREM 2 levels with NPC1 clinical phenotypes (a) age of neurological onset, >20 years excluded from the analysis. (b) annual severity increment score, (c) 17-domain NPC neurological severity score, and (d) 5-domain NPC neurological severity score.

Additional Figure 7: Spearman correlation of NPC1 serum GPNMB levels with NPC1 clinical phenotypes (a) 17-domain NPC neurological severity score, and (b) 5-domain NPC neurological severity score.

Additional Figure 8: Expression levels of proteins based on gender: there were no gender-based differences in the expression level of (a) TREM2, (b) HSD17B14, (c) NPY, (d) Cathepsin L, (e) CCL18, (f) AgRP, and (g) BDNF. An unpaired two-tailed t-test was used to evaluate the differences between males and females.

Additional Figure 9: Spearman correlation of NPC1 serum BDNF levels with NPC1 clinical phenotypes (a) annual severity increment score, (b) 17-domain NPC neurological severity score, and (c) 5-domain NPC neurological severity score.

Additional Figure 10: Spearman correlation of NPC1 serum HSD17B14 levels with NPC1 clinical phenotypes (a) age of neurological onset, (b) individuals with age of neurological onset >20 years were excluded from the analysis, (c) annual severity increment score, (d) 17-domain NPC neurological severity score, and (e) 5-domain NPC neurological severity score.

Additional Figure 11: Spearman correlation of NPC1 serum CCL18 levels with NPC1 clinical phenotypes. Serum CCL18 levels did not correlate with (a) age of neurological onset, (b) individuals with age of neurological onset >20 years were excluded from the analysis, (c) Annual severity increment score, (d) 17-domain NPC neurological severity score, and (e) 5-domain NPC neurological severity score.

Additional Figure 12: Spearman correlation of NPC1 serum AgRP levels with NPC1 clinical phenotypes. Serum AgRP levels did not correlate with (a) age of neurological onset, (b) individuals with age of neurological onset >20 years were excluded from the analysis, (c) Annual severity increment score, (d) 17-domain NPC neurological severity score, and (e) 5-domain NPC neurological severity score.

Additional Figure 13: Spearman correlation of NPC1 serum NPY levels with NPC1 clinical phenotypes. Serum NPY levels did not correlate with (a) age of neurological onset, (b) individuals with age of neurological onset >20 years were excluded from the analysis, (c) Annual severity increment score, (d) 17-domain NPC neurological severity score, and (e) 5-domain NPC neurological severity score.

Additional Figure 14: Spearman correlation of NPC1 serum Cathepsin L levels with NPC1 clinical phenotypes. Serum Cathepsin L levels did not correlate with (a) age of neurological onset, (b) individuals with age of neurological onset >20 years were excluded from the analysis, (c) Annual severity increment score, (d) 17-domain NPC neurological severity score, and (e) 5-domain NPC neurological severity score.

Additional Figure 15: Graphical representation of the annotation of significantly increased proteins in NPC1 detected in the Human Protein Atlas (a) increased with adjusted log_2_FC ≥ 0.5 and < 1, and (b) increased with adjusted log_2_FC < 0.5.

Additional Figure 16: Graphical representation of the annotation of significantly decreased proteins in NPC1 detected in the Human Protein Alas (a) decreased with adjusted log_2_FC ≤ 1.5 and > -2, (b) decreased with adjusted log_2_FC ≤ -1 and >-1.5, (c) decreased with adjusted log_2_FC ≤ -0.5 and >- 1, and (d) decreased with adjusted log_2_FC > - 0.5.

Additional Figure 17: GSEA functional enrichment of differentially abundant proteins in NPC1 versus control comparison (a) Biological process, (b) Molecular function; and NPC1 miglustat versus no miglustat comparison (c) Biological process, (d) Molecular function. Y-axis: GO molecular function terms; X-axis (Gene Ratio): Proportion of genes associated with each term; Dot size (Count): Number of genes in that category; Color (p.adjust): Statistical significance (red = more significant)

**Additional Tables**

Additional Table 1 (ST1): Sample IDs, age, and sex information for the NPC1 individuals and healthy pediatric controls used in the PEA screening. Information for the NPC1 individuals: Miglustat status, duration of miglustat, Age of Neurological onset, 5- Domain Neurological Severity Score, 17- domain Neurological Severity Score, Annual Severity Increment Score (ASIS), cDNA variants and protein variants. Sample IDs start with ‘NPC’ and ‘C’ for NPC1 individuals and healthy pediatric control samples, respectively.

Additional Table 2 (ST2): Differentially abundant proteins in NPC1 versus control comparisons.

Additional Table 3 (ST3): Differentially abundant proteins in NPC1 samples receiving miglustat versus NPC1 samples not receiving miglustat.

Additional Table 4 (ST4): Integration of NPC1 disease and miglustat treatment effects on the serum proteome.

Additional Table 5 (ST5): Spearman correlation of NPC1 clinical parameters with proximal extension assay NPX values.

Additional Table 6 (ST6): List of overlapping proteins between NSS17 and NSS5; 28 proteins positively correlated with both NSS17 and NSS5, and 336 proteins negatively correlated with both NSS17 and NSS5.

Additional Table 7 (ST7): Correlation of NPX values with the age of neurological onset with statistical models.

Additional Table 8 (ST8): Correlation of NPX values with the 17-domain NPC NSS with statistical models.

Additional Table 9 (ST9): Annotations of differentially abundant proteins in NPC1 with the Human Protein Atlas.

Additional Table 10 (ST10): List of differentially altered proteins in NPC1 with brain-specific expression (tau >0.90).

**Column Name Description for Additional Tables**

1. Assay – Protein names
2. GeneID – ensemble ID
3. Panel – Olink panel
4. Uniprot – Uniprot ID
5. OlinkID – unique Olink ID for the gene
6. Effect - Model term (e.g., NPC1 status, Age, Sex, Interaction)
7. pes - Partial eta squared; proportion of variance explained by that effect (0–1)
8. p.value – Raw P value for the Fold change
9. Adjusted_pval – FDP corrected P values
10. Threshold – Threshold signifies if the Adjusted P value has crosse the significance criteria of adjusted p value < 0.1
11. Formula- Model used for adjusting the fold change values while adjusting for covariates. In the formula wherever applicable:
12. **NSS_17_c:** Continuous, mean‑centered disease burden score (NSS‑17).
13. **NSS_17_c × Age_c:** Age‑dependent modification of burden effect.
14. **Age_c / ANO_c:** Mean‑centered covariates for age and age of neurological onset.
15. **ANO_c × Age_c:** Age‑dependent modification of onset effect.
16. **NSS_17_c × Miglustat:** Interaction between burden and treatment status.
17. **Sex:** Sex covariate.
18. **Miglustat:** Treatment status covariate.
19. **Age_c:** Mean‑centered age covariate.
20. **ANO_c:** Continuous, mean‑centered age of neurological symptom onset.
21. Log2FC – Log 2 fold change in the NPX values relative to controls
22. Estimate/ Adjusted Log2FC – Esitmated marginal means aka emmeans, represents adjusted log₂ fold-change
23. Assay_Panel – Protein name along with the panel. Since there were many proteins that were present in multiple panels, this new column created a unique
24. sig_npc_vs_ctrl – Significant in NPC1 versus Control comparison
25. sig_npc_mig_vs_npc_no_mig – Significant in NPC1 miglustat treated versus Miglustat non- treated samples
26. rank_abs - Rank based on |estimate| across proteins
